# Post-streptococcal Hemophagocytic Lymphohistiocytosis in an Immunocompetent Adult

**DOI:** 10.7759/cureus.106139

**Published:** 2026-03-30

**Authors:** Halah Z Keramane, Sarine Tahmazian, Yaseen Belkadi, Vivek Soi, Diane L Levine

**Affiliations:** 1 Internal Medicine, Wayne State University School of Medicine, Detroit, USA; 2 Internal Medicine, Henry Ford Health System, Detroit, USA; 3 Internal Medicine, Kentucky College of Osteopathic Medicine, Pikeville, USA; 4 Nephrology, Henry Ford Health System, Detroit, USA

**Keywords:** group a streptococcal pharyngitis, hemophagocytic lymphohistiocytosis (hlh), hemophagocytic syndrome (hs), hemophagocytic syndrome in infections, secondary hlh, undifferentiated shock

## Abstract

Secondary hemophagocytic lymphohistiocytosis (HLH) is a rare and life-threatening hyperinflammatory syndrome that can occur after acute infection, predominantly caused by viruses. Due to its nonspecific presentation and clinical overlap with conditions like sepsis, HLH is frequently underrecognized in adults. We present the case of an immunocompetent adult admitted with undifferentiated shock, which rapidly progressed to multi-organ failure. Upon further investigation, the patient was diagnosed with HLH, and a comprehensive infectious workup revealed streptococcal pharyngitis as the precipitating cause. This case emphasizes the importance of recognizing HLH in the differential diagnosis in immunocompetent adults with pharyngitis presenting with shock, as early recognition is crucial for timely intervention and improved outcomes. Additionally, we review the current literature to characterize the reported infectious causes of HLH in immunocompetent adults in order to highlight the importance of having a low index of suspicion and conducting a comprehensive infectious workup.

## Introduction

Hemophagocytic lymphohistiocytosis (HLH) is a rare hyperinflammatory disorder characterized by uncontrolled immune activation and has been described as both an inherited and sporadic syndrome [1,2]. The cause of secondary HLH is multifactorial, with one underlying trigger often predominating, although the precise pathophysiological mechanisms remain incompletely understood. According to the threshold model described by Brisse et al. [3], multiple factors - including genetic susceptibility, baseline inflammation, immunosuppression, and infectious triggers - act cumulatively until a critical threshold is reached, resulting in uncontrolled and fulminant inflammation. Individuals with secondary HLH may harbor partial genetic defects, which impair but do not completely abolish the ability to regulate immune responses. In adults, more severe disease may also be associated with variants in HLH-related genes. Consequently, diverse etiologies may converge on a common endpoint of hyperinflammation, making identification of the initial trigger challenging in some cases [2]. The inherited form, primary HLH, has been linked to mutations in *PRF1*, *UNC13D*, *STX11*, and *STXBP2* [2]. Secondary HLH, in which there is no germline mutation in genes that regulate lymphocytic function, is triggered by external factors that cause immune dysregulation [1]. Some precipitating factors include infections, malignancies, and immune deficiencies [4]. Of the identified infectious triggers for secondary HLH, viruses are the most common, with Epstein-Barr virus (EBV) being the most frequent inciting agent [1]. The clinical presentation of HLH is variable and nonspecific, and patients often present with high fever, hepatosplenomegaly, and pancytopenia [5]. Less common presentations include jaundice, lethargy, seizures, and end-organ dysfunction [5]. Pertinent laboratory findings include markedly elevated lactate dehydrogenase, increased ferritin, coagulopathy, and specific markers of end-organ damage [5]. HLH is diagnosed based on the HLH-2004 diagnostic criteria, which require either genetic confirmation or fulfillment of at least five of eight clinical and laboratory features, reflecting uncontrolled immune activation [1,3]. Even with aggressive treatment, this disorder has a poor prognosis [5,6]. Thus, timely diagnosis is essential to improving patient outcomes, and emphasis must be placed on recognition of common and uncommon triggers [5]. To our knowledge, there have not been any reported cases of HLH secondary to streptococcal pharyngitis in an immunocompetent adult. We present this case to document this etiology in the literature, describe the presentation and diagnostic workup, and emphasize the importance of a low threshold for suspicion and timely intervention.

## Case presentation

An adult male patient, in his late forties, presented to the emergency department with five days of fatigue, chills, and poor oral intake. Confusion, nausea, vomiting, diarrhea, and dizziness began two days prior to admission. He had a history of obesity, well-controlled type 2 diabetes mellitus, and obstructive sleep apnea. His home medications included empagliflozin and acetaminophen. A few weeks prior to admission, he was diagnosed with a mild upper respiratory infection and was treated symptomatically. His travel and social history were unremarkable, and he had no recent sick contacts.

Upon presentation, he was tachycardic and tachypneic but normotensive and saturating greater than 92% oxygen on room air. Physical examination was significant for lethargy, respiratory distress, abdominal tenderness, and swollen tonsils with white, patchy exudates. Generalized lymphadenopathy was present, but splenomegaly was not. The remainder of the exam was normal. A complete blood count was remarkable only for thrombocytopenia. Renal function tests showed acute kidney injury with an elevated creatinine of 4.43 mg/dL as compared to his baseline of 0.66 mg/dL (reference range 0.6-1.13 mg/dL). Serum lactic acid was also elevated at 4.3 mmol/L (reference range <2.1 mmol/L). A chest X-ray showed pulmonary edema. Computerized tomography of the head, abdomen, and pelvis was unrevealing. Rapid antigen test for *Streptococcus pyogenes* was found to be positive.

The patient subsequently developed undifferentiated shock with worsening mental status, respiratory distress requiring intubation, and refractory hypotension requiring vasopressors. He was started on empiric vancomycin and ceftriaxone for suspected sepsis pending cultures. Renal function continued to decline, leading to worsening acidosis and initiation of sustained low-efficiency dialysis. Liver function tests showed hypoalbuminemia at 3.0 g/dL (reference range 3.7-4.8 g/dL), an aspartate aminotransferase level of 1,969 IU/L (reference range <45 IU/L), and an alanine aminotransferase level of 715 IU/L (reference range <52 IU/L). Workup for Hepatitis B and C, hemolysis, and autoimmune processes were negative, as were urine drug screen and blood toxicology. Blood and urine cultures were also negative. Labs revealed diffuse organ dysfunction, markedly elevated serum ferritin, elevated triglycerides, and elevated soluble interleukin-2 (IL-2) receptor at 23,903.1 pg/mL (reference range 175.3-858.2 pg/mL). Due to his fever, elevated ferritin, elevated triglycerides, and elevated soluble IL-2, the patient met diagnostic criteria for HLH with an H-score of 182, indicating a diagnosis probability of about 80%. Serial laboratory values are outlined in Table 1.

**Table 1:** Serial laboratory values during hospitalization for hemophagocytic lymphohistiocytosis

	Reference Values	Day 1	Day 2	Day 3
Red blood cells (M/µL)	4.40-6.00 M/µL	4.91	4.58	3.75
White blood cells (K/µL)	3.8-10.6 K/µL	22.3	19.6	15.4
Platelets (K/µL)	150-450 K/µL	54	82	75
Ferritin (ng/mL)	24-336 ng/mL	65,766	13,728	9,657
Triglycerides (mg/dL)	<200 mg/dL	828	974	779
Fibrinogen activity (mg/dL)	200-450 mg/dL	301	347	225

Further infectious workup was negative for severe acute respiratory syndrome coronavirus 2 (SARS-CoV-2), influenza A virus, influenza B virus, hantavirus, cytomegalovirus (CMV), EBV, human immunodeficiency virus (HIV), herpes simplex virus type 1 (HSV-1), varicellazoster virus (VZV), *Histoplasma capsulatum*, *Blastomyces dermatitidis*, *Anaplasma phagocytophilum*, *Leptospira interrogans*, *Treponema pallidum*, and *Borrelia burgdorferi*. Empiric antibiotics were then discontinued, and the patient was started on intravenous dexamethasone and *Pneumocystis jirovecii* pneumonia prophylaxis with trimethoprim-sulfamethoxazole. He had a gradual improvement in liver and kidney function over his hospital course, with recovery of his mental status and orientation on day 10 of hospitalization. He was discharged on a dexamethasone taper with follow-up in the hematology and oncology clinic.

## Discussion

**Figure 1:**
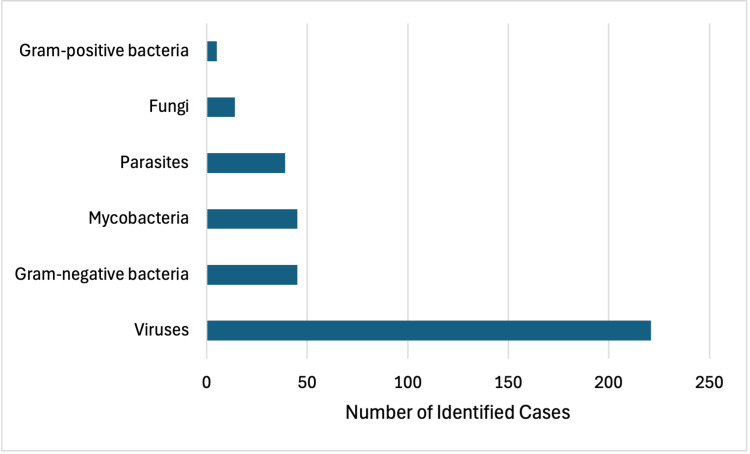
Reported infectious etiologies of hemophagocytic lymphohistiocytosis

Bacterial causes of HLH are less common than viral etiologies, accounting for around 9% of reported cases [7]. Using the PubMed database, we conducted a review of reported infectious triggers of HLH. The search terms were used: “hemophagocytic lymphohistiocytosis infection adult”. The results were filtered for case reports between 2004 and August 2025. Articles were included based on the following criteria: (1) the patient was diagnosed with HLH, (2) a causative infectious agent was identified, (3) the patient was an adult above the age of 18, and (4) the patient was not immunocompromised at symptom onset. The search was conducted manually, and article titles and abstracts were screened. Duplicate articles were excluded. The search yielded 962 results. Cases not pertaining to HLH, without an infectious etiology, pediatric cases, and immunocompromised cases were excluded. Of 369 cases, only five identified a gram-positive bacterium as the causative agent. There were two reported cases secondary to *Staphylococcus aureus*, one of which resulted from dialysis-related methicillin-resistant *S. aureus* bacteremia and one that resulted from superinfection after viral illness [8,9]. Of the two cases caused by *Streptococcus pneumoniae*, one occurred during a six-day course of prednisone therapy and another described superinfection after a viral illness [10,11]. The last case occurred secondary to *Enterococcus faecalis* infection in a patient with a reported history of alcohol abuse [12]. Patients were most often treated with steroids, and some were additionally treated with intravenous immunoglobulin (IVIG). Our search identified no reported cases secondary to *S. pyogenes* infection. A summary of the findings is listed in Figure 1.

A case secondary to Group G streptococcal endocarditis has also been reported [13]. Most bacterial cases we identified occurred secondary to mycobacterium or gram-negative bacterium. Our results suggest that cases due to gram-positive bacterium are less frequent but have been reported [8-12]. The results may have been limited by selection bias and the inclusion of case reports alone, which limits the generalizability of our findings. However, our search gives an estimate of the relative prevalence of infectious causes and emphasizes the importance of a comprehensive workup when evaluating HLH etiology due to the wide spectrum of precipitating infectious causes.

In 1991, the Histiocyte Society proposed diagnostic criteria encompassing various findings to estimate the probability of HLH in adults, requiring at least five out of eight criteria to be met to support the diagnosis [14]. These criteria include: (1) fever, (2) splenomegaly, (3) cytopenia affecting two or more blood cell lineages, (4) hypertriglyceridemia and/or hypofibrinogenemia, and (5) evidence of hemophagocytosis in bone marrow, spleen, or lymph nodes [15]. The following criteria were added in 2004: (6) ferritin greater than or equal to 500 μg/L, (7) low or absent natural killer cell activity, and (8) high soluble IL-2 receptor alpha chain [14]. The diagnostic criteria are met if five out of eight criteria are fulfilled or there is a molecular diagnosis consistent with HLH [14].

Because of the variation in the underlying cause, presentation, and treatment response, treatment algorithms focus on supportive therapy, immunosuppression, and treatment of the underlying cause [1,4]. After stabilization, antibiotics or cytotoxic therapy, in the case of underlying malignancy, should be started [4]. For persistent or severe inflammatory states, steroids and prophylactic therapy for opportunistic infections are frequently initiated [4]. Recent preclinical research has suggested a potential role for monoclonal antibodies and other biologic therapies, but their efficacy and safety have not been definitively established [1,4].

## Conclusions

To the best of our knowledge, our patient represents a rare reported case of HLH secondary to *S. pyogenes* pharyngitis in an immunocompetent adult. While causality cannot be definitively established from a single case, this report suggests that even mild streptococcal infections may act as a potential trigger for HLH. Therefore, a high index of suspicion should be maintained in patients who present with undifferentiated shock refractory to empiric antibiotic therapy. The rapid progression to multi-organ failure emphasizes the severity of this condition and the importance of timely intervention. Early diagnosis may be facilitated by application of established diagnostic criteria and heightened awareness of the hyperinflammatory features characteristic of HLH.
